# Comprehensive Viral Detection and Profiling of Plasma Cell‐Free RNA in Patients With Suspected Hemophagocytic Lymphohistiocytosis

**DOI:** 10.1002/jmv.71122

**Published:** 2026-09-01

**Authors:** Yuto Fukuda, Kazuhiro Horiba, Masanori Hashino, Shinji Kawabe, Hiroki Miura, Yoshiki Kawamura, Makito Tanaka, Takako Suzuki, Yuka Torii, Hideki Muramatsu, Yoshiyuki Takahashi, Tetsushi Yoshikawa, Jun‐ichi Kawada

**Affiliations:** ^1^ Department of Pediatrics Nagoya University Graduate School of Medicine Nagoya Japan; ^2^ Laboratory of Microbial Genomics, Pathogen Genomics Center, National Institute of Infectious Diseases Japan Institute for Health Security Tokyo Japan; ^3^ Department of Infection and Immunology Aichi Children's Health and Medical Center Aichi Japan; ^4^ Department of Pediatrics Fujita Health University School of Medicine Toyoake Aichi Japan; ^5^ Department of Pediatrics Fujita Health University Okazaki Medical Center Okazaki Japan

**Keywords:** cell‐free RNA, Hubei reo‐like virus, metagenomic next‐generation sequencing, mitochondrial RNA

## Abstract

Hemophagocytic lymphohistiocytosis (HLH) is a severe, rapidly progressive disease. While viral infection is considered a common etiology of pediatric HLH, specific causative viruses other than the Epstein‐Barr virus (EBV) have been rarely identified. This study utilized metagenomic next‐generation sequencing (NGS) to identify potential causative pathogens in plasma samples from 17 pediatric patients with suspected HLH. Additionally, one case each of confirmed EBV‐ and cytomegalovirus (CMV)‐associated HLH was analyzed for methodological validation. Plasma cell‐free RNA (cfRNA) profiling was performed using NGS data to assess the host transcriptome response. Significant viral reads of human herpesvirus‐6B, human herpesvirus‐7, and Hubei reo‐like virus (HRLV) 14 were detected using metagenomic NGS in one patient each. Plasma cfRNA profiles from five patients with viral infection (including EBV and CMV) were compared to those of 14 patients without viral infection. By comparing the two patient groups, 1053 differentially expressed genes were identified. The gene ontology (GO) term of “adaptive immune response” (GO: 0002250) was significantly enriched among upregulated genes in the virus‐positive group. Furthermore, an isolated cluster consisting specifically of mitochondrial RNAs, was identified in the upregulated genes of the virus‐positive group. Using metagenomic NGS, several candidate viral pathogens were identified in patients with suspected infection‐related HLH. The viral genome of HRLV 14, previously undetected in human clinical samples, was identified in one patient. The results from plasma cfRNA profiling suggest that mitochondrial RNAs may reflect the underlying pathogenesis of virus‐associated HLH and have potential utility as disease biomarkers.

## 1 Introduction

Hemophagocytic lymphohistiocytosis (HLH) is a severe, rapidly progressive disease that can be fatal. HLH is characterized by uncontrolled hypercytokinemia, persistent fever, cytopenia, and hemophagocytosis caused by activated macrophages in the bone marrow. HLH is classified into two categories: primary HLH, resulting from genetic immune abnormalities, and secondary HLH, occurring subsequent to infections, malignant lymphomas, or autoimmune diseases [1]. While various types of viral infections are associated with HLH, Epstein‐Barr virus (EBV) is considered the most common viral etiology of HLH [1, 2, 3]. A nationwide survey of HLH in Japan showed that EBV‐associated HLH accounted for 53.1% of all HLH cases [4]. Conversely, specific causative pathogens other than EBV have rarely been identified in patients with suspected infection‐related HLH. Despite recent advances in viral detection methodologies, including multiplex PCR, the identification of viral agents in patients with HLH remains a challenge in clinical practice.

Metagenomic next‐generation sequencing (NGS) has been used for pathogen detection for over a decade [5, 6]. We previously utilized metagenomic NGS for the comprehensive detection of viral pathogens across various clinical specimens, including those from patients with encephalitis, pneumonia, and myocarditis [7, 8, 9, 10]. NGS enables rapid and comprehensive identification of viral pathogens in a single assay. Additionally, human‐derived reads excluded during pathogen detection can be used for host transcriptomic analysis [11]. However, reports describing the simultaneous analysis of pathogen and host transcriptomes using a single sample are limited.

Plasma cell‐free RNA (cfRNA) is actively released from dying cells, circulating cells, and cells from various tissues [12]. Circulating cfRNAs are abundant in plasma, and their profiles can be analyzed by NGS. Although the biological significance of cfRNA remains incompletely understood, it has emerged as a novel biomarker for cancer diagnosis and disease monitoring [12, 13]. Furthermore, plasma cfRNA profiles can characterize organ injury and host–pathogen interactions in patients with inflammatory syndromes and infectious diseases [14, 15, 16]. Wang et al. showed that plasma cfRNA analysis can simultaneously monitor immune responses and pneumonia‐related microbial communities in patients with severe COVID‐19 [17]. Therefore, plasma cfRNA analysis using NGS is a promising approach for the diagnosis and pathological evaluation of infectious diseases.

In this study, we performed metagenomic NGS to identify the causative pathogens in patients with suspected HLH using plasma samples. Subsequently, the plasma cfRNA profiles of patients with detected viral infections were compared with those of patients in whom viral pathogens were not identified.

## 2 Materials and Methods

### 2.1 Patients and Samples

We retrospectively enrolled 19 patients diagnosed with or suspected of having infection‐related HLH at Nagoya University Hospital or Fujita Health University Hospital between 2016 and 2023. In this study, patients who met five or more of the eight criteria described in the HLH‐2004 guideline [18] were classified as having HLH, whereas those who met 2–4 criteria were classified as having suspected HLH. Malignancies, rheumatic diseases, and immune deficiencies were excluded as potential causes of HLH‐like symptoms based on laboratory findings and clinical courses in these patients. The etiology remained undetermined at the time of the initial treatment in 17 of the 19 patients. Two patients for whom EBV or cytomegalovirus (CMV) DNA had already been detected by real‐time PCR in whole blood were included to evaluate the diagnostic performance of metagenomic NGS. Plasma samples were collected in the acute phase and stored at –30°C until further use.

### 2.2 Library Preparation, Sequencing, and Metagenomic NGS Data Analysis

Clinical specimens were processed by enzymatic treatment with MagNA Pure Bacteria Lysis Buffer and Proteinase K (Roche, Mannheim, Germany), followed by mechanical disruption using ZR Bashing Bead Lysis Tubes (Zymo Research, Irvine, CA, USA). Bacterial lysis buffer and bead beating were used to maximize nucleic acid recovery for comprehensive pathogen detection, including bacteria and fungi. Nucleic acids were then extracted using the Maxwell RSC miRNA Tissue Kit (Promega, Madison, WI, USA) according to the manufacturer's instructions. DNA libraries were prepared from RNase‐treated nucleic acids using a QIAseq FX DNA Library Kit (Qiagen, Hilden, Germany). RNA libraries were prepared from DNase‐treated nucleic acids using a Zymo‐Seq RiboFree Total RNA Library Kit (Zymo Research). Sequencing was performed using the NextSeq. 2000 system (Illumina, San Diego, CA, USA) with a 2 × 150 bp paired‐end protocol. Metagenomic analysis and pathogen detection were performed using the PATHDET pipeline and pathogen detection criteria as previously described in our study [11]. Transcriptome analysis of the RNA sequencing data was conducted according to the protocols described in the same report. The TCC package was used to generate volcano plots and heat maps for visualization of the transcriptome analysis.

### 2.3 Pathway Analysis

Pathway and molecular function analyses of significantly differentially expressed genes, defined as those upregulated > 2.0‐fold with *p* < 0.05, were performed using Metascape (https://metascape.org) [19].

### 2.4 Real‐Time PCR

Real‐time PCR for EBV, CMV, human herpesvirus (HHV)‐6B, and HHV‐7 was performed using a QuantStudio Real‐Time PCR System (Applied Biosystems, Foster City, CA, USA) as previously described [20, 21].

## 3 Results

### 3.1 Patient Characteristics

The demographic and laboratory characteristics of the patients are presented in Table 1. Alongside high fever of ≥ 38.5°C, cytopenia affecting ≥ 2 of 3 lineages and hyperferritinemia of ≥ 500 µg/L were observed in 10 and 15 patients, respectively.

**Table 1:** Baseline characteristics.

Characteristics	All patients (*n* = 19)	Virus‐positive group (*n* = 5)	Virus‐negative group (*n* = 14)	*p* value^a^
Age, years, median (IQR)	9 (2–12)	6 (1.5–12)	9.5 (6.5–13)	0.587
Male, *n* (%)	10 (53)	2 (40)	7 (50)	0.7
Laboratory, median (IQR)				
White blood cell (10^3^/μL)	3200 (1500–5770)	1200 (990–2700)	4300 (2600–6100)	0.16
Neutrophil (10^3^/μL)	900 (570–1790)	700 (390–2550)	1100 (700–1800)	0.67
Platelets (10^4^/μL)	8.5 (5.7–14.9)	6.4 (3.3–7.9)	12.7 (10–12.4)	**0.03**
Hemoglobin (g/dL)	11.0 (9.9–12.2)	10.0 (9.5–11.1)	12.0 (10.0–12.4)	0.15
AST (U/L)	160 (70–460)	300 (80–660)	160 (60–350)	0.86
ALT (U/L)	140 (30–280)	100 (30–190)	140 (50–410)	0.28
LDH (U/L)	610 (460–820)	850 (630–1100)	530 (460–740)	0.45
Ferritin (ng/mL)	860 (640–5540)	4440 (2600 –7660)	790 (640–3800)	0.41
Triglyceride (mg/dL)	190 (110–220)	200 (120–210)	160 (110–220)	1.0
Fibrinogen (mg/dL)	220 (150–290)	190 (130–240)	220 (160–300)	0.41

Abbreviations: AST, aspartate aminotransferase; ALT, alanine aminotransferase; IQR, interquartile range; LDH, lactate dehydrogenase.

^a^ Continuous variables and dichotomous data were compared using Mann–Whitney U test and *χ* ^2^ test, respectively. bold *p* values indicate satistical significance *p* < 0.05.

### 3.2 Detection of Viruses From Plasma Samples

**Figure 1:**
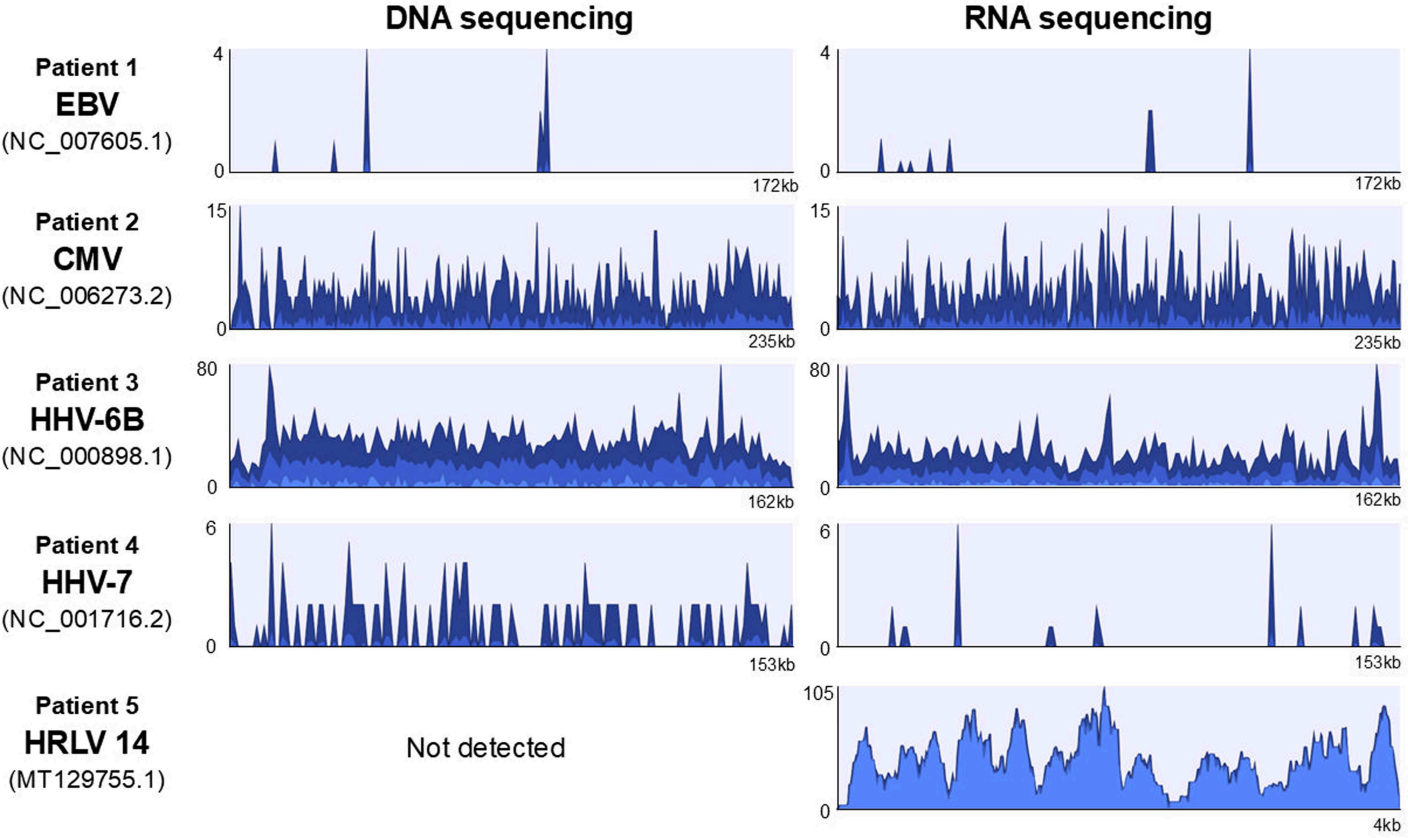
Coverage plots of viral genomes detected in plasma samples. Sequencing reads obtained from each plasma sample were aligned to the reference viral genome using the NCBI accession numbers specified for each case. Light blue, blue, and very light blue denote the minimum, mean, and maximum coverage depths, respectively. CMV, cytomegalovirus; EBV, Epstein‐Barr virus; HHV‐6B, human herpesvirus 6B; HHV‐7, human herpesvirus 7; HRLV 14, Hubei reo‐like virus 14.

Significant viral reads were detected in the plasma of five patients by DNA and/or RNA metagenomic sequencing. These included two patients in whom EBV or CMV was detected by real‐time PCR of whole blood (patients #1 and #2, respectively). The genome coverage plots for EBV and CMV are shown in Figure 1. A summary of the viruses detected in these five patients is shown in Table 2. No significant bacterial reads were detected in any of the patients.

**Table 2:** Detection of virus‐derived reads from the plasma of patients.

Patient #	Age/sex	DNA sequencing		RNA sequencing		PCR results (copies/mL)
		Detected virus	Read count (total reads)	Detected virus	Read count (total reads)	
**1**	5 y/F	EBV	12 (5,503,439)	EBV	68 (6,213,936)	EBV (7500, whole blood)
**2**	1 m/M	CMV	2253 (23,382,334)	CMV	10,050 (69,173,136)	CMV (112,000, whole blood)
**3**	1 y/M	HHV‐6B	20,483 (49,237,990)	HHV‐6B	43,179 (133,552,710)	HHV‐6B (1,115,700, whole blood)
**4**	12/M	HHV‐7	198 (27,428,607)	HHV‐7	30 (8,860,450)	HHV‐7 (6,100, plasma)
**5**	15 y/F	Not detected	0 (71,505,548)	Hubei reo‐like virus 14	1258 (46,911,273)	Not done

*Note:* Metagenomic sequencing and PCR were performed using same specimen in each patient.

Abbreviations: CMV, cytomegalovirus; EBV, Epstein‐Barr virus; HHV‐6B, human herpesvirus 6B, HHV‐7, human herpesvirus 7.

HHV‐6B reads were detected in the plasma of patient #3 (a 1‐year‐old boy) by DNA and RNA sequencing (Figure 1 and Table 2). The patient presented with a high fever and met the diagnostic criteria for HLH‐2004. The patient's condition improved after treatment with dexamethasone and cyclosporine A. Pathogenic gene variants/mutations in familial HLH‐related genes, including *PRF1*, *UNC13D*, and *STX11*, were not detected. High levels of HHV‐6B DNA (1,115,700 copies/mL) were detected in one whole blood sample using real‐time PCR. A decrease in the HHV‐6B DNA load to 490 copies/mL was observed 5 months later; therefore, endogenous HHV‐6 (chromosomally integrated HHV‐6) was ruled out.

HHV‐7 reads were detected in the plasma of patient #4 (a 12‐year‐old boy) by DNA and RNA sequencing (Figure 1 and Table 2). The presence of HHV‐7 DNA in plasma was also confirmed by real‐time PCR (Table 2). The patient presented with clinical features suggestive of HLH, including high fever, bicytopenia, and hyperferritinemia. The patient's condition was managed with antibiotic therapy, and the fever resolved on the seventh day of hospitalization. Specific HLH‐directed therapy was not initiated because the cytopenia and hyperferritinemia resolved spontaneously.

Hubei reo‐like virus 14 (HRLV 14) reads were detected in patient #5 (a 15‐year‐old girl) by RNA sequencing (Figure 1 and Table 2). The presence of HRLV 14 in plasma could not be verified due to insufficient sample volume. The patient had been receiving cyclosporine A since the age of 8 years for suspected systemic juvenile idiopathic arthritis. The patient presented with a high fever, pancytopenia, and hyperferritinemia, which raised concerns regarding HLH. The patient was treated with prednisolone; consequently, her clinical symptoms and laboratory parameters improved.

### 3.3 Transcriptome Analysis of cfRNA in Plasma

**Figure 2:**
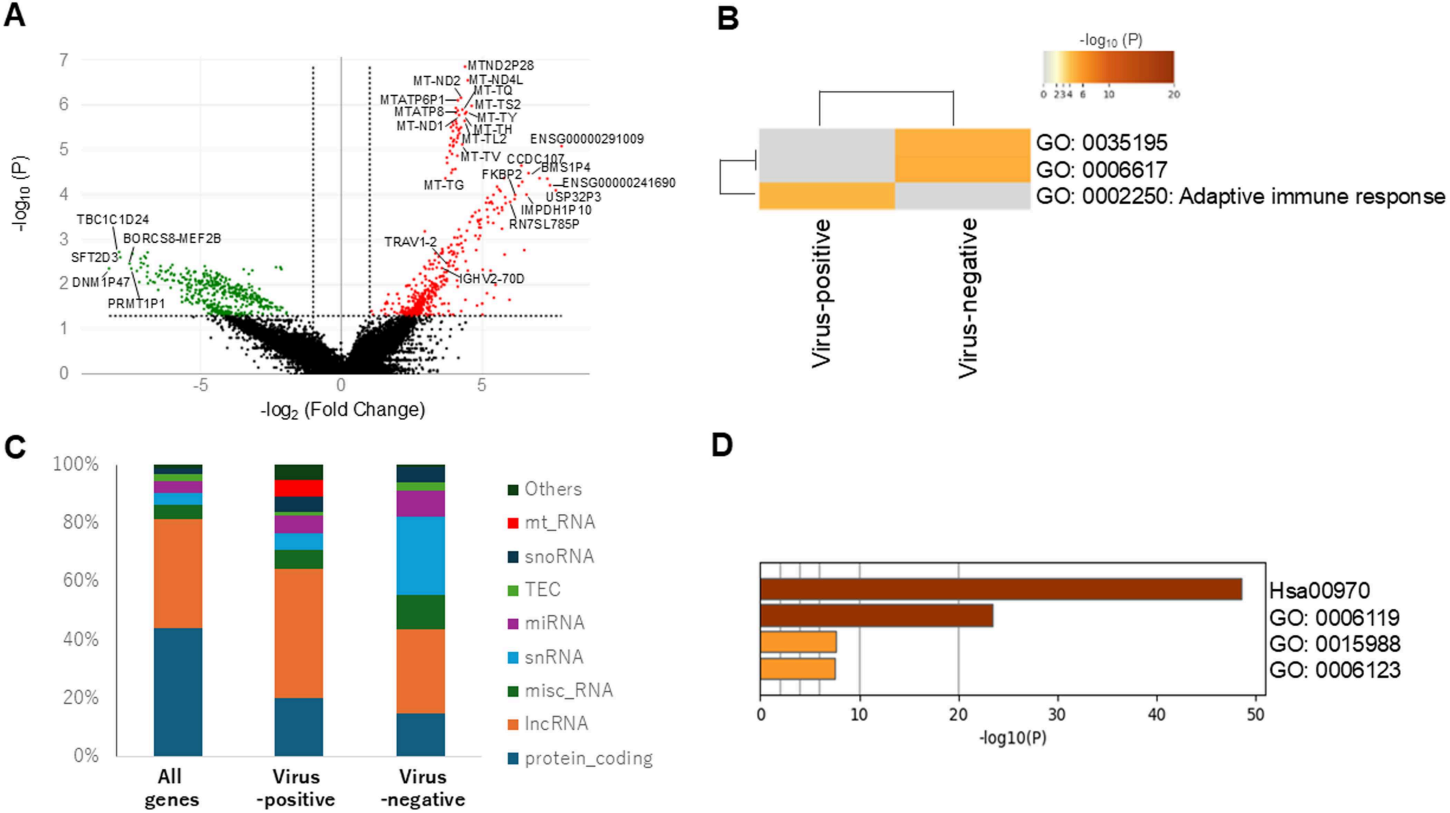
Transcriptome analysis of cell‐free RNA in plasma. (A) Volcano plot showing differentially expressed genes between the virus‐positive and ‐negative groups. Red and green dots indicate upregulated genes in the virus‐positive and ‐negative groups, respectively. The horizontal and vertical dashed lines indicate a *p* value threshold of 0.05 and a fold change threshold of 2.0, respectively. (B) Heatmap of enriched terms of upregulated genes in the virus‐positive and ‐negative groups, colored by *p* values, generated using Metascape. GO: 0035195, signal sequence recognition and miRNA‐mediated post‐transcriptional gene signaling; GO00066617, SRP‐dependent cotranslational protein targeting to membrane. (C) The average read percentage of different types of RNAs in each group. (D) The enriched terms of upregulated mitochondrial RNAs in the virus‐positive group. hsa00970, aminoacyl‐tRNA biosynthesis; GO:0006119, oxidative phosphorylation; GO: 0015988, energy‐coupled proton transmembrane transport, against electrochemical gradient; GO: 0006123, mitochondrial electron transport, cytochrome c to oxygen.

Human‐derived reads from RNA sequencing data were used for host transcriptomic analysis. Among an average of 30,652,201 total RNA sequencing reads per sample, an average of 28,045,543 reads (91.5%) were mapped to the host‐derived RNA. Plasma cfRNA transcriptome profiles of five patients with detected viral infection (patients # 1–5, virus‐positive group) were compared with those of 14 patients without virus detection (virus‐negative group). A comparison of the demographic and laboratory characteristics of the two groups is presented in Table 1. In total, 1053 differentially expressed genes (DEGs) were identified between the two patient groups. A volcano plot depicting the DEGs between the two groups is shown in Figure 2A. The top 20 upregulated genes in the virus‐positive and ‐negative groups are shown in Supporting Information S1: Tables S1 and S2, respectively. Long non‐coding RNA (ENSG00000291009) and ubiquitin‐specific peptidase 32 pseudogene 3 were the most and second‐most upregulated genes in the virus‐positive group, whereas dynamin 1 pseudogene 47 and TBC1 domain family member 24 were the most and second‐most upregulated genes in the virus‐negative group. All DEGs were enriched in the corresponding gene ontology (GO) categories using the MetaScape software. The “adaptive immune response” (GO: 0002250) term was enriched among the genes upregulated in the virus‐positive group (Figure 2B). The upregulated genes associated with the adaptive immune response category are listed in Supporting Information S1: Table S3. Conversely, SRP‐dependent co‐translational proteins targeting the membrane (GO00066617), signal sequence recognition, and miRNA‐mediated post‐transcriptional gene signaling (GO0035195) were enriched among the upregulated genes in the virus‐negative group (Figure 2B).

### 3.4 Cell‐Free Mitochondrial RNAs in Patients With Detected Viral Infection

In the volcano plot shown in Figure 2A, an isolated cluster of cell‐free mitochondrial RNAs was identified among the upregulated genes in the virus‐positive group. The proportions of the different RNA types in each group are shown in Figure 2C. Mitochondrial RNAs comprised 5.8% of the upregulated genes in the virus‐positive group, whereas they represented 0.05% and 0% of all the detected genes and upregulated genes in the virus‐negative group, respectively. The upregulated mitochondrial RNAs that formed this cluster are listed in Supporting Information S1: Table S4. The most and second‐most enriched GO categories among the mitochondrial RNAs were aminoacyl‐tRNA biosynthesis (hsa00970) and oxidative phosphorylation (GO:0006119) (Figure 2D).

## 4 Discussion

Viral infections, particularly EBV, are the most common triggers of HLH [1]. However, the identification of viral etiologies other than EBV infection remains a challenge in clinical practice. HHV‐6B and HHV‐7, which belong to the *Roseolovirus* genus of the herpesvirus subfamily, were identified in one patient each in this study. Although HHV‐6B is considered a trigger for HLH, the number of reported cases is substantially lower than that of EBV‐associated HLH, particularly in immunocompetent individuals without underlying conditions [22, 23]. The HHV‐6B‐positive patient in this cohort was a previously healthy boy with no genetic abnormalities associated with primary HLH. Nevertheless, the patient fulfilled the diagnostic criteria for HLH and required immunomodulatory therapy. HHV‐7 has rarely been reported as a causative agent of HLH. Recently, Wang et al. reported an adult case of familial HLH in which HHV‐7 was identified in the blood using metagenomic NGS [24]. The HHV‐7‐positive case of suspected HLH in this study was a 12‐year‐old boy who demonstrated spontaneous resolution. Notably, HHV‐7 detected in plasma may reflect viral reactivation following HLH‐like symptoms.

In this study, a high number of HRLV 14 reads were detected in a patient receiving cyclosporine A therapy. HRLV is a double‐stranded RNA virus recently identified through metagenomic analysis of invertebrate specimens collected in China [25], and HRLV 14 genome sequences have been identified in Diptera species in Australia [26]. HRLV belongs to the genus Orbivirus, which comprises viruses capable of infecting a diverse range of arthropods and vertebrate hosts [26]. This is the first case in which HRLV genome was detected in a human clinical sample. Therefore, further investigations are warranted to elucidate the pathogenic potential of HRLV.

In addition to metagenomic analysis to identify viral pathogens, plasma cfRNA profiling was performed to investigate the host response in patients with viral infections. Notably, many of the top upregulated genes in the virus‐positive group were pseudogenes or related to novel proteins/transcripts, which limited the identification of a definitive pattern. Given the limitations of short‐read sequencing and the high sequence similarity between pseudogenes and their parental genes, these findings should be interpreted with caution, as some may reflect ambiguous mapping rather than true pseudogene‐specific expression. Conversely, genes belonging to the adaptive immune response GO category, including T cell receptor genes and immunoglobulin genes, were significantly upregulated in the virus‐positive group (Supporting Information S1: Table S3), which is considered a reasonable result reflecting the host immune response to the virus. These results suggest that plasma cfRNA profiling is a valuable tool for analyzing the pathogenesis of viral infectious diseases.

Notably, cell‐free mitochondrial genes were upregulated in patients with viral infections (Figure 2A,C). Mitochondria play critical roles in various antiviral responses, including type I interferon signaling [27]. Furthermore, the activation of mitochondrial cell death pathways, characterized by apoptosis and pyroptosis, is observed during viral infection to restrict viral replication [27, 28]. The death of virus‐infected cells also acts as a signal to alert other immune cells about the infection. In contrast, the contribution of mitochondrial cell death to the severity of HLH has been demonstrated in a murine model [29]. The release of mitochondrial RNA into the circulation is a potential consequence of extensive cell death and mitochondrial damage. These mitochondrial components may trigger hyper‐inflammatory responses, which are characteristic of HLH [30]. Therefore, the detection of cell‐free mitochondrial RNA is a reasonable finding in the context of virus‐associated HLH. Although further validation is required, plasma mitochondrial RNAs may serve as biomarkers for severe viral infections, including virus‐associated HLH.

To the best of our knowledge, this is the first study to use metagenomic NGS to investigate viral pathogens and perform cfRNA profiling in patients with suspected HLH. However, this study had several limitations. First, this was a retrospective study with a small sample size. Second, metagenomic sequencing was performed exclusively on plasma samples. Consequently, HLH‐like symptoms may have been triggered by viral infection in some patients in whom viral DNA or RNA was not detected in plasma. Moreover, establishing a causal relationship between detected viruses and clinical symptoms was challenging. Third, cfRNAs were not validated using other methods, such as reverse transcription PCR.

## Author Contributions

Y.F., K.H., and J.K. conceived and designed the study. Y.F., K.H., and M.H., conducted experiments. S.K., H.M., Y.K., M.T., T.S., Y.T., and T.Y. collected the clinical samples and data. Y.F., K.H., and J.K. analyzed the data and wrote the manuscript.

## Ethics Statement

The study design and purpose were approved by the Institutional Review Board of Nagoya University Hospital (IRB number: 2015‐0236) and Fujita Health University Hospital (IRB number: HM24‐260). Informed consent was obtained from all patients or their guardians.

## Conflicts of Interest

The authors declare no conflicts of interest.

## Supporting information


Supporting File

## Data Availability

The data that support the findings of this study are available on request from the corresponding author.
